# Digital Peer-Supported Self-Management Intervention Codesigned by People With Long COVID: Mixed Methods Proof-of-Concept Study

**DOI:** 10.2196/41410

**Published:** 2022-10-14

**Authors:** Hayley Wright, Andrew Turner, Stuart Ennis, Carol Percy, Garry Loftus, Wendy Clyne, Gabriela Matouskova, Faith Martin

**Affiliations:** 1 Centre for Intelligent Healthcare Coventry University Coventry United Kingdom; 2 Department of Cardiac Rehabilitation University Hospitals Coventry & Warwickshire NHS Trust Coventry United Kingdom; 3 Warwick Clinical Trials Unit Warwick Medical School University of Warwick Coventry United Kingdom; 4 Atrium Health Centre for Exercise and Health Coventry United Kingdom; 5 School of Psychological, Social and Behavioural Sciences Faculty of Health and Life Sciences Coventry University Coventry United Kingdom; 6 Hope For The Community Community Interest Company Enterprise Hub Coventry United Kingdom; 7 Peninsula Medical School University of Plymouth Plymouth United Kingdom

**Keywords:** long COVID, self-management, peer support, digital intervention, goal setting, psychological, physical, cognitive, intervention, United Kingdom, UK, efficacy, COVID-19

## Abstract

**Background:**

There are around 1.3 million people in the United Kingdom with the devastating psychological, physical, and cognitive consequences of long COVID (LC). UK guidelines recommend that LC symptoms be managed pragmatically with holistic support for patients’ biopsychosocial needs, including psychological, emotional, and physical health. Self-management strategies, such as pacing, prioritization, and goal setting, are vital for the self-management of many LC symptoms.

**Objective:**

This paper describes the codevelopment and initial testing of a digital intervention combining peer support with positive psychology approaches for self-managing the physical, emotional, psychological, and cognitive challenges associated with LC. The objectives of this study were to (1) codesign an intervention with and for people with LC; (2) test the intervention and study methods; (3) measure changes in participant well-being, self-efficacy, fatigue, and loneliness; and (4) understand the types of self-management goals and strategies used by people with LC.

**Methods:**

The study used a pre-post, mixed methods, pragmatic, uncontrolled design. Digital intervention content was codeveloped with a lived-experience group to meet the needs uncovered during the intervention development and logic mapping phase. The resulting 8-week digital intervention, Hope Programme for Long COVID, was attended by 47 participants, who completed pre- and postprogram measures of well-being, self-efficacy, fatigue, and loneliness. Goal-setting data were extracted from the digital platform at the end of the intervention.

**Results:**

The recruitment rate (n=47, 83.9%) and follow-up rate (n=28, 59.6%) were encouraging. Positive mental well-being (mean difference 6.5, *P*<.001) and self-efficacy (mean difference 1.1, *P*=.009) improved from baseline to postcourse. All goals set by participants mapped onto the 5 goal-oriented domains in the taxonomy of everyday self-management strategies (TEDSS). The most frequent type of goals was related to activity strategies, followed by health behavior and internal strategies.

**Conclusions:**

The bespoke self-management intervention, Hope Programme for Long COVID, was well attended, and follow-up was encouraging. The sample characteristics largely mirrored those of the wider UK population with LC. Although not powered to detect statistically significant changes, the preliminary data show improvements in self-efficacy and positive mental well-being. Our next trial (ISRCTN: 11868601) will use a nonrandomized waitlist control design to further examine intervention efficacy.

## Introduction

“Long COVID” (LC) is the term commonly used when symptoms continue or develop after acute COVID‐19. It includes ongoing symptomatic COVID‐19 (from 4 to 12 weeks) and post–COVID‐19 syndrome (12 weeks or more), where symptoms cannot be explained by an alternative diagnosis [1]. There are around 1.3 million people (2% of the population) with LC in the United Kingdom [1]. Of those, 40% report symptoms lasting at least 1 year and 64% report symptoms that adversely affect their daily activities [1]. The most common symptoms are fatigue (51%), loss of smell (37%), breathlessness (36%), and difficulty concentrating (28%)—also known as “brain fog” [1]. Mental well-being can also be impacted through the distress of living with a long-term health condition [2].

Symptoms vary within and between individuals and in severity. Patients often feel dismissed by medical professionals and seek validation elsewhere, such as peer support groups [3,4]. People with LC can also experience social stigma and invalidation in the wider community, further adding to the burden of coping [5-7]. LC has devastating psychological, physical, and cognitive consequences that disrupt lives and livelihoods. There are currently 90 specialist post–COVID-19 clinics across the United Kingdom, but there is wide local variation in referral rates, waiting times, and access across demographic groups [8]. Many services were suspended due to staff shortages following the rise in cases of the Omicron variant [9].

Since the first wave of the pandemic, people with LC have sought virtual peer support for managing their symptoms, driven by the lack of formal support in the early days of the pandemic [4]. In response to the pandemic generally, there has been a rapid and essential growth in the provision of digital health care for long-term conditions to allow remote care [10,11], and patients are more motivated, accepting, and familiar with digital technologies for health care and social connection [12]. The main digital resource for people with LC in the United Kingdom is “Your COVID Recovery” [13], a comprehensive National Health Service (NHS) website providing general symptom management advice and limited professional support. However, this broad resource lacks the social connection that patients desire [4], and evaluations of patient experiences of the intervention have yet to be published.

UK COVID-19 guidelines recommend that LC symptoms be managed pragmatically with holistic support for patients’ biopsychosocial needs, including psychological, emotional, and physical health [14]. Activities should be *paced* and only performed if they do not worsen symptoms, such as breathlessness or fatigue [15,16]. Self-management strategies, such as pacing, prioritization, and goal setting, are vital for the self-management of many LC symptoms, such as fatigue [15]. However, there is still no NHS-endorsed, evidence-based, peer-supported self-management program for the multifaceted issues faced by people with LC.

NHS England recommends that patients with LC symptoms be treated in accordance with the national post–COVID-19 syndrome pathway, which encourages self-care through engaging patients in techniques such as specific, measurable, achievable, relevant, and timely (SMART) *goal setting* and *peer support* [17]. Goal setting is a core component of self-management of long-term conditions and can support patients to change behaviors. Setting goals empowers patients to maintain or improve management of their own physical, psychological, and social health. SMART goal setting helps to break down a desired achievement (ie, goal) into manageable chunks (ie, action plan), and SMARTER goal setting incorporates “enjoyable” and “reward” elements [18]. However, peer support and goal setting have not yet been evaluated in LC self-management programs. Improvement is nonlinear, and recovery to pre–COVID-19 levels of health and fitness is not guaranteed, so evaluation of goal setting is imperative.

This paper describes the codevelopment and testing of a digital intervention combining peer support with evidence-based behavior change techniques for self-managing the physical, emotional, psychological, and cognitive challenges associated with LC. The Hope Programme for Long COVID provides a proactive, timely solution in the most urgent public health crisis in a generation. We repurposed an existing digital self-management intervention—the digital Hope Programme—which has shown postcourse improvements in depression, anxiety, and mental well-being in multiple participant groups, such as cancer survivors [19,20], people with multiple sclerosis [21], and parents of children with autism [22]. The Hope Programme for Long COVID combines positive psychology and cognitive behavioral approaches, providing essential psychological support for people to live with the physical symptoms of LC and is delivered by trained facilitators.

This paper describes the codesign process and pre- and postprogram changes in mental well-being, self-efficacy, fatigue, and loneliness. In this study, we aimed to codesign and test a bespoke self-management intervention with and for people with LC. Specifically, the objectives of the study were to:

- Codesign bespoke intervention content specifically for LC.
- Test the intervention and study methods to inform future study design (calculate recruitment and follow-up rates and summarize demographics and symptoms of the sample).
- Measure changes in key indicators of well-being, self-efficacy, fatigue, and loneliness to signal efficacy of the intervention (calculate the difference in pre- and postprogram outcomes).
- Examine goal-setting data to better understand the types of self-management goals and strategies used by participants (perform a thematic analysis of the types of goals set by participants).

## Methods

### Study Design

The wider project was led by Hope For The Community (H4C) Community Interest Company, with the aim to empower people across Coventry, Warwickshire, and Rugby (CWR) to self-manage their health and well-being and to develop social connections and peer support opportunities. The study reported here adopted a pre-post, mixed methods, pragmatic, uncontrolled design to codesign and test a digital self-management intervention for people with LC.

### Participants

Adults with self-reported LC were eligible to enroll into the Hope Programme for Long COVID. Participants were informed that they must be over the age of 18 years and have access to an internet-enabled device to access the intervention content. No further exclusion criteria were applied. The intervention delivery partner (H4C) recruited people to enroll into the intervention using a range of social media, posters, flyers, and mailshots in the CWR area. Subsequently, those who had enrolled were contacted by email and asked whether they wanted to also participate in the research. Nonparticipation in the research had no impact on their receipt of the intervention. The email invitation to the study contained the necessary participant information sheet, online consent forms, and a link to complete baseline research questionnaires (via Qualtrics survey software). The intervention was hosted on the H4C digital platform.

### Intervention

The digital Hope Programme for Long COVID was cocreated to enable a rapid response to the ongoing public health crisis in the wake of the COVID-19 pandemic. The Hope Programme for Long COVID shares the same underlying theoretical framework as the digital Hope Programme, which has been described in detail elsewhere [19,20]. In summary, the Hope Programme for Long COVID focuses on strengths rather than deficits and uses group curative factors, including instilling hope, universality (realizing you are not alone), and altruism [23]. By sharing successful coping strategies, the peer support of the group achieves something greater than the sum of its parts. Through positive psychology, the Hope Programme for Long COVID cultivates an upward spiral of positive emotions [24] to improve well-being and coping. Hope and gratitude—2 important concepts in positive psychology—are core themes embedded within the Hope Programme for Long COVID. They are addressed through the experience of group curative factors [23] and through specific activities, such as goal setting and feedback, identification of strengths, and gratitude diaries [25-27]. The Hope Programme for Long COVID content was codesigned by people with LC and health care professionals who support them.

#### Codesign Methods

We conducted 3 workshops: 2 workshops with a total of 8 people with LC and 1 workshop with 6 professionals involved in LC health care. In addition, 1-to-1 sessions were completed with 2 people with LC who could not join the group sessions. A total of 16 participants took part in the codesign activities.

**Figure 1:**
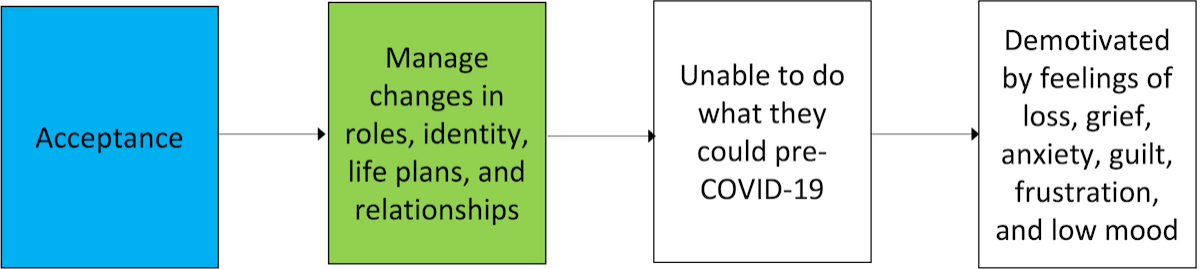
Extract from logic map created during co-development of the Hope Programme for Long COVID.

A conversational and informal approach was used to facilitate discussion and open expression of views. Workshops were conducted online via Zoom. Participants were asked to reflect upon what “antecedents” caused the problem statement “People living with long COVID face many challenges in doing self-management” and then were asked to talk about what causes the antecedents, working back each time something was mentioned by asking “What causes this?” This process is based on elements of the antecedent target measurement process described by Renger and Hurley [28]. Responses were captured by the researcher in a logic map (see Figure 1, for example), noting each chain of antecedents that lead to the problem. Reading from right (white boxes) to left (green box), the map shows that antecedents (root causes) of challenges in engaging in self-management stem from negative feelings caused by a shift in abilities. The green box shows the need for support to manage change, and the blue box represents the target behavior of acceptance, which is addressed by the intervention. Participants were invited to add further antecedents or details throughout the discussion and on checking the logic map with the researcher.

A rapid literature search of the research databases PsycInfo, Cumulative Index to Nursing and Allied Health Literature (CINAHL), and Medline identified literature relating to support or self-management needs for people with LC. LC is a new condition, and there is a relative paucity of research, so we also searched for literature on chronic fatigue syndrome (CFS) and myalgic encephalomyelitis (ME), which share many of the key symptoms of LC (eg, fatigue, breathlessness, brain fog). Titles and abstracts of papers were assessed for relevance, prioritizing reports of recent systematic reviews of needs and interventions in LC. Research papers were then examined to ensure the needs identified by our stakeholder groups were supported by the research evidence. Reports of existing interventions for conditions with similar symptoms helped identify components that may be effective in supporting people with LC. This information was used to ensure an underlying evidence base for the foci and components of the Hope Programme for Long COVID.

The digital Hope Programme content [19-21,29] was repurposed to reflect the needs and individual lived experience of people with LC. New content was developed to meet the needs uncovered during the intervention development phase and logic mapping. A cognitive psychologist (author HW) led the codesign of the module addressing the management and understanding of brain fog, and a clinical psychologist (author FM) codeveloped new material on acceptance, self-compassion, and managing fatigue. Evidence-based breathing and physical exercises were provided by an exercise physiologist (author SE). All the intervention content was developed specifically to address the needs of people with LC, with goal setting embedded within each module. All modules were reviewed by the multidisciplinary team and patients with lived experience of LC, prior to testing the full program.

Key features of the Hope Programme for Long COVID, as identified by stakeholders and supported by the literature, include:

- Peer support has been integral to patients’ recovery and understanding of COVID-19 throughout the pandemic [4], helping patients feel less lonely and isolated. Our workshop participants asked for this essential source of support to be included in the Hope Programme for Long COVID. Participants can interact with peers in an optional weekly Hope Café via Zoom or through online discussion forums and email. They are encouraged to support each other’s goals and gratitude posts by posting likes and comments, where feedback strengthens group cohesiveness and a sense of belonging [23]. The Hope Programme for Long COVID is asynchronous, so it can be accessed as and when required, and text content is presented in concise sections measured against readability indices, such as the Gunning Fog Index [30]. These design features support participants in pacing their learning and progress through the course at their own speed.
- Facilitated delivery: Two exercise specialists from Atrium Health were trained in health coaching and motivational interviewing to become Hope Programme facilitators. They were available to answer participants’ questions and stimulate discussions between participants throughout the Hope Programme for Long COVID.
- Evidence-based guidelines relating to recovery and rehabilitation for COVID-19 are still emerging and can be inconsistent at best [31]. Evidence-based information about how to self-manage symptoms of LC and signposting to further information are provided by the NHS Your COVID Recovery web-based resource for UK patients [13]. However, during codesign, our stakeholders reported that patients experiencing fatigue may be overwhelmed by the amount of information to read. To mitigate these limitations, the Hope Programme for Long COVID provides bite-sized, evidence-based information, embedded in interactive videos, diagrams, and discussion forums, which is consolidated in the processes of social networking and peer support.
- Across our workshops, stakeholders were unanimous in their support for pacing [32] for people with LC. Many had experienced the “boom-and-bust cycle,” where overexertion in times of high energy (boom) leads to periods of extreme fatigue (bust). Pacing materials and activities were codesigned with a clinical psychologist (FM) with experience of supporting patients in an NHS LC clinic. Participants described how fatigue can be caused by physical, mental, or emotional exertion. In collaboration with exercise physiologists (SE and team), we developed bespoke, expert-delivered physical activity content for the Hope Programme for Long COVID, with a clear focus on pacing and participants staying within their “energy envelope.” Prioritization and goal setting [29,33] are central to self-management approaches and are core elements of pacing. These are all vital techniques for fatigue management in LC [15].

Access to the Hope Programme for Long COVID is unrestricted, sits outside of the NHS and so does not require participants to have a clinical diagnosis or referral, and is free of charge at the point of access to users. Weekly program content and activities are summarized in Table 1.

**Table 1:** Weekly topics and activities.

Session	Examples of content, in addition to weekly goal setting	Examples of exercises and activities
Week 1: Introduction: Instilling Hope	Welcome and introductions The benefits of positive emotions The power of gratitude Self-compassion Personalized goal setting Video: How to Set Achievable Goals Dates for live sessions	Self-management tools: Interactive gratitude diary SMARTER^a^ goal setting Rectangle breathing exercise for LC^b^ Self-test: How are you feeling?
Week 2: Long COVID Symptoms	Fatigue management, including the boom-and-bust cycle, prioritizing, planning, and pacing What is brain fog, and what can we do to help ourselves? Forum topics: sharing experiences of managing fatigue and brain fog Further resources and links to LC information and support	Self-management tools: Interactive gratitude diary SMARTER goal setting and goal feedback Activity and fatigue diary Pacing planner
Week 3: Managing Stress	Coping with unhelpful thinking patterns Understanding and managing stress Self-compassion and acceptance Mindfulness for stress management and meditation Video: How to Be Kind to Yourself Further resources and links (eg, videos, podcasts, and websites) to self-compassion, mindfulness, and stress management	Self-management tools: Interactive gratitude diary SMARTER goal setting and goal feedback Guided relaxation and meditation Self-care checklist (worksheet)
Week 4: Communication	Communication skills and tips for talking with health professionals, your employer, and your family Preparing for difficult conversations Asking for and accepting help Compassion for worries Further resources and links (eg, videos, podcasts, and websites) to LC support groups	Self-management tools: Interactive gratitude diary SMARTER goal setting and goal feedback “Ask the Expert” live session Self-test: How are you feeling?
Week 5: Sleep and Mindfulness	How does COVID-19 affect our sleep? Tips for sleeping better Tips to aid relaxation Introduction to mindfulness, meditation, and relaxation	Self-management tools: Interactive gratitude diary SMARTER goal setting and goal feedback Raisin meditation Breathing exercises Live mindfulness meditation session via Zoom
Week 6: Move Better, Feel Better	Keeping active with LC Postexertional malaise Tips for getting active Eating well for physical and mental health Managing changes to taste and smell after COVID-19	Self-management tools: Interactive gratitude diary SMARTER goal setting and goal feedback Quiz: What contributes to happiness? Activity: gentle stretches and exercises
Week 7: Happiness and Strengths	LC and effects on mood Happiness and hope Identifying your character strengths Understanding how using your strengths can lead to a more fulfilling life Video: The Science of Character Strengths Managing setbacks Tips for authentic happiness, managing setbacks, and keeping going	Self-management tools: Interactive gratitude diary SMARTER goal setting and goal feedback Video: guided meditation activity
Week 8: Moving Forward With Hope	Hopes and dreams Doing something for yourself Planning pleasant activities Keeping in touch with peers Review of the program Feedback Staying hopeful	Self-management tools: Interactive gratitude diary SMARTER goal setting and goal feedback Self-test: How are you feeling?

^a^SMARTER: specific, measurable, achievable, relevant, time-based, enjoyable, rewarded.

^b^LC: long COVID.

#### Outcome Measures

##### Sociodemographics

Participants were asked their name, email address, gender, date of birth, postcode, occupation, highest level of education attained, and details of formal treatment received for COVID-19–related symptoms.

##### Symptom Logging

The COVID-19 Yorkshire Rehabilitation Screening Tool (C19-YRS [34]) screens for the most common complications after COVID-19 (eg, breathlessness, fatigue, cognitive problems) and rates the severity or significance of each symptom on a scale of 0-10 (0=no impact to 10=significant impact).

The logic map from the cocreation activities set out what issues were to be addressed by the intervention, and this study measured the impact of the intervention on these issues. Validated, standardized questionnaires were used to measure changes in key factors commonly affected by LC. The intervention components were designed to:

- Increase positive mental well-being
- Increase confidence to self-manage (self-efficacy)
- Reduce fatigue
- Reduce feelings of loneliness

##### Positive Mental Well-being

The Warwick-Edinburgh Mental Wellbeing Scale (WEMWBS [35]) has previously been used in research on people with long-term conditions [19,20]. The items (questions) are worded positively and refer to function and feelings. For each item, there is a choice of 5 responses ranging from 1 for “none of the time” to 5 for “all the time.” The responses are summed to obtain a total score ranging from 14 to 70, where a higher score indicates a higher level of mental well-being. A score of 40 or less is indicative of probable depression, and a change of 3 or more in the total score represents a minimally important level of change. Two versions of WEMWBS were used in this study: The full measure was used at baseline and end of the intervention, and the shorter 7-item version of the WEMWBS (score range 7-35) was embedded within the Hope Programme for Long COVID in sessions 1, 4, and 7, allowing participants to monitor their own mental well-being throughout the course.

##### Self-efficacy

The 6-item Self-Efficacy for Managing Chronic Disease scale (SEMCD6 [36]) provides a robust measure of participants’ confidence to self-manage their symptoms of LC. The questions relate to participants’ confidence that they can keep issues relating to their condition from interfering with daily life. Responses to each of the 6 questions are on a 10-point scale, ranging from 1 for “not at all confident” to 10 for “totally confident.” The total ranges from 6 to 60, and the self-efficacy score is calculated as the mean score across all items (eg, total/6), with a range of 1-10. There are no recommended cut-off scores for this scale, but data from a population of 489 individuals with chronic disease show an average self-efficacy score of 5.2 [37].

##### Fatigue

The Fatigue Severity Scale (FSS [38]) measures fatigue and its effect on activities and lifestyle in patients with a variety of conditions. It comprises 9 questions answered on a 7-point scale, ranging from 1 for “strongly disagree” to 7 for “strongly agree.” The questions relate to how fatigue interferes with certain activities, and severity is indicated by the 7-point scale. Summed answers give a total score ranging from 9 to 63, and mean scores range from 1 to 7. Higher scores indicate higher fatigue severity. Research suggests that a mean score of 4 or more is indicative of fatigue [39].

##### Loneliness

The University of California, Los Angeles (UCLA) Loneliness Scale, Version 3 [40], is a 3-item scale to assess loneliness and is recommended by the Office of National Statistics [41]. The 3 questions are as follows:

- How often do you feel that you lack companionship?
- How often do you feel left out?
- How often do you feel isolated from others?

The response scale is scored as 1 for “hardly ever,” 2 for “some of the time,” and 3 for “often.” The total score is the sum of all items (ranging from 3 to 9), where higher scores indicate greater feelings of loneliness.

### Sample Size

A target sample size of N=40 was deemed adequate for this study, informed by evidence that sample sizes of 24-50 are sufficient to estimate the key parameters of an efficacy trial [42]. Attendance on the Hope Programme for Long COVID was capped at 60 people to allow manageable facilitation and communication between participants.

### Analysis

Descriptive statistics were used to summarize recruitment and retention rates. All statistical data analyses were conducted using IBM SPSS Statistics version 26. The sample was not fully powered to detect significance in the outcome measures, but we presented changes in scores and preliminary *P* values to demonstrate the potential of the intervention (ie, to signal efficacy) and provide data on which to base power calculations for a larger study of efficacy. The level of statistical significance was set at *P*<.05. Outcomes were analyzed using paired-sample *t* tests. We reported the means with SDs and effect size estimates.

Goal-setting data were analyzed using deductive content analysis, with coding derived from the taxonomy of everyday self-management strategies (TEDSS) [43]. TEDSS focusses on self-management strategies for persistent symptoms, such as those that have become synonymous with LC (eg, fatigue), that lead to reduced physical and cognitive function. The taxonomy categorizes self-management strategies for conditions that are typically difficult to mitigate and have a significant impact on everyday life [43], which again are typical of the fluctuating symptoms of LC. The types of goals set by participants were coded according to the 5 goal-oriented domains:

- Activity strategies
- Internal strategies
- Social interaction strategies
- Health behavior strategies
- Disease-controlling strategies

All goals were coded by 1 researcher, and a random selection of 25% of the data was second-coded by another researcher. An interrater reliability score (Cohen κ) of <.70 was used as a cut-off to indicate all data should be second-coded.

### Ethical Considerations

All participants received a Participant Information Sheet describing the purpose and anticipated outcomes of the research, including participants’ right to decline to take part or withdraw from the research at any time, and data processing in accordance with the Data Protection Act of 2018. Participants were informed that their data would be treated confidentially and would be anonymized by the use of a randomly generated participant identification number in Qualtrics. The digital consent form required participants to confirm that they had read and understood the Participant Information Sheet and agreed to their anonymized data being used in research publications and dissemination. All statements had to be agreed upon before participants could progress into the study. Upon completion of pre- and postprogram questionnaires, participants were entered into a prize draw for a chance to win a £50 (US $52.87) voucher. The research was reviewed and approved by the Black Country Research Ethics Committee (IRAS ID: 283172) and the Coventry University Research Ethics Committee (P106036).

## Results

### Recruitment and Follow-up

**Figure 2:**
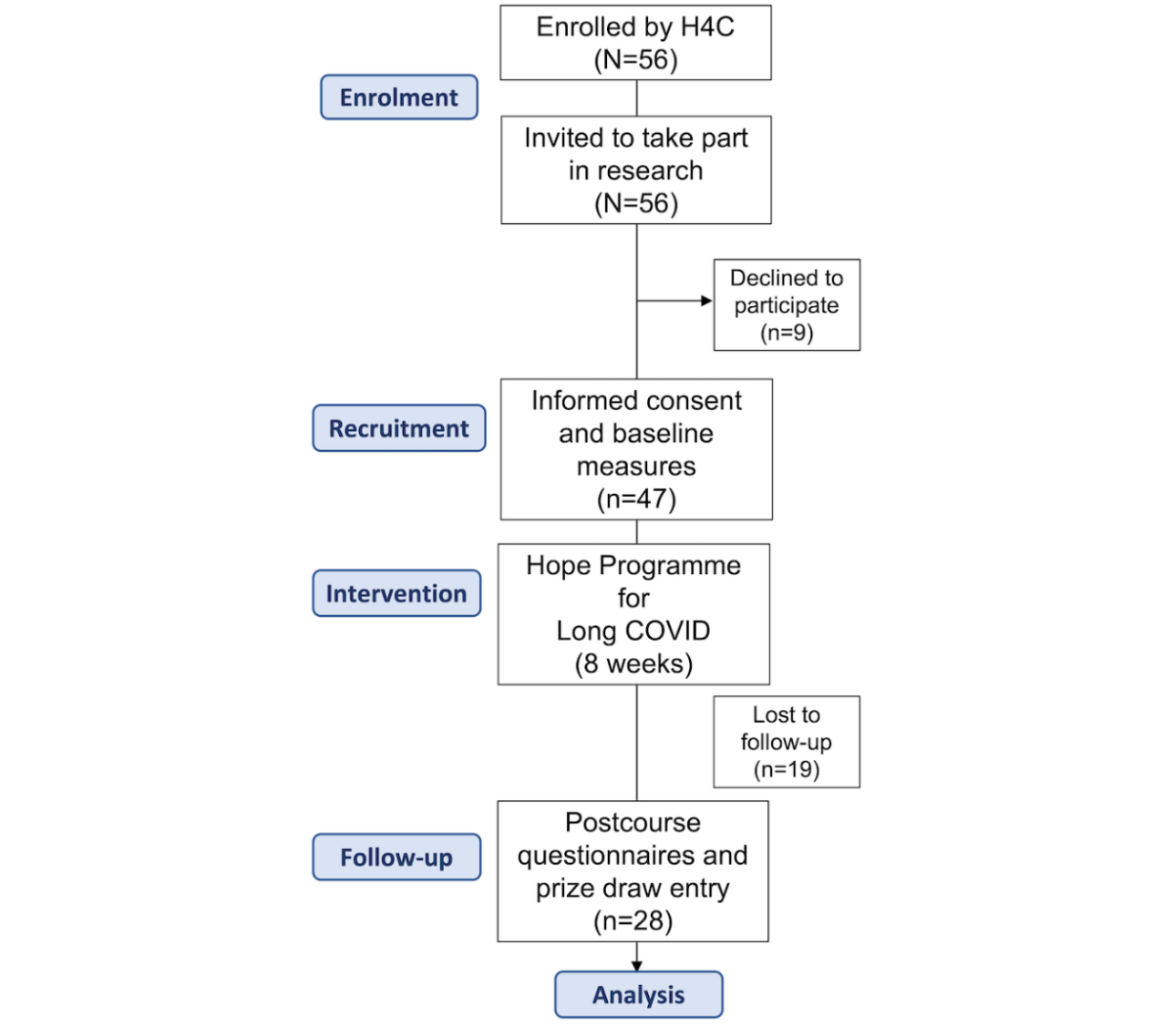
Participant flow through the study. H4C: Hope For The Community.

A total of 56 people enrolled into the Hope Programme for Long COVID, of which 47 (recruitment rate 83.9%) consented to take part in the study and provided baseline data. A total of 28 participants completed the postcourse questionnaires, giving a follow-up rate of 59.6% (see Figure 2 for participant flow).

### Sociodemographics and Symptoms

Sociodemographic data describing the characteristics of the sample and the severity of the impact of LC symptoms are shown in Tables 2 and 3, respectively. Baseline data suggested that the characteristics of the sample were similar to the general UK population with LC [1]. The mean age of this group was 48.8 years (SD 11.0), 39 (83%) were female, 42 (91.3%) described themselves as White British, 36 (78.2%) were employed, and 36 (76.6%) had reduced their working hours due to their COVID-19–related illness.

All participants (n=47, 100%) reported fatigue at baseline, and the majority reported cognitive issues, including problems with concentration (n=46, 98%), memory (n=44, 94%), and communication (n=34, 72%), such as word finding and following a conversation. On average, COVID-19–related symptoms had persisted for longer than a year across the sample. The severity or impact of symptoms was rated highest for fatigue, usual daily activities (eg, shopping, household tasks), and cognitive communication (all rated 7/10, on average), followed by breathlessness on walking up stairs and pain (both rated 6/10, on average). A summary of severity scores is shown in Table 3.

**Table 2:** Sample characteristics at baseline.

Characteristics		Baseline (N=47)
Age^a^ (years), mean (SD)		48.8 (11.0)
Female, n (%)		39 (83.0)
**Ethnicity^a^, n (%)**		
	White British	42 (91.3)
	Asian British	3 (6.5)
	Prefer not to say	1 (2.2)
Post-16 education, n (%)		37 (78.7)
**Employment status^a^, n (%)**		
	Full time, paid	22 (47.8)
	Part time, paid	14 (30.4)
	Unemployed/retired/stay-at-home parent	10 (21.7)
Cut work hours due to LC^b^, n (%)		36 (76.6)
At least 1 long-term condition prior to LC^c^, n (%)		30 (73.2)
LC symptom duration in days^a^, mean (SD)		377.3 (171.8)
Positive COVID-19 test or diagnosis, n (%)		34 (72.3)
Hospitalized with acute COVID-19, n (%)		2 (4.3)
Accessed NHS^d^ LC clinic, n (%)		19 (40.4)

^a^N=46 due to missing data.

^b^LC: long COVID.

^c^N=41 due to missing data.

^d^NHS: National Health Service.

**Table 3:** LC^a^ symptoms and mean severity scores across the sample at baseline.

LC symptoms/impact (N=47)		Severity score (range 0-10), mean (SD)
Fatigue		7.0 (2.1)
Cognitive/communication		6.6 (2.4)
Impacting daily activities		6.9 (2.4)
**Breathlessness**		
	Walking up stairs	5.7 (2.8)
	On dressing	3.3 (2.7)
	At rest	2.4 (2.2)
Personal care		3.0 (3.1)
Pain/discomfort		5.5 (2.4)
Throat sensitivity		5.2 (2.0)
Voice changes		5.1 (1.7)
Swallowing difficulties		5.0 (1.7)
Reduced appetite		3.5 (2.6)
Reduced mobility		5.0 (2.7)
General health		5.1 (1.9)
Anxiety		5.1 (2.8)
Depression		4.1 (3.4)

^a^LC: long COVID.

### Health and Well-being

Outcome data from participants completing both baseline and postcourse questionnaires are summarized in Table 4. Paired *t* tests measured statistically significant changes in pre-post well-being scores to signal efficacy of the intervention. Positive mental well-being (WEMWBS) scores increased by 6.5 points, on average, from baseline to postcourse (*P*<.001). In addition, the Short-Form Warwick-Edinburgh Mental Wellbeing Scale (SWEMWBS) was embedded within the Hope Programme for Long COVID in weeks 1, 4, and 7 (see Table 5). Repeated-measures ANOVA showed a significant difference in SWEMWBS scores across the 3 time points (*F*_1.4,22.1_=20.2, *P*<.001). Bonferroni-corrected comparisons showed a significant increase in scores between week 1 and week 4 (*P*<.001) and between week 1 and week 7 (*P*=.001) but no difference in scores between week 4 and week 7 (*P*=.25). Self-efficacy also improved from baseline to postcourse (mean difference 1.1, SD 1.9, *P*=.01). Fatigue severity scores were unchanged from baseline to postcourse (mean difference 0.4, SD 1.6, *P*=.25). Similarly, loneliness scores remained unchanged from pre- to postcourse (mean difference 0.2, SD 1.8, *P*=.60).

**Table 4:** Summary of baseline and postcourse well-being measures.

Measure	Baseline score (N=28), mean (SD)	Postcourse score (N=28), mean (SD)	Difference, mean (SD)	*t* (df)	*P* value	Effect size (Cohen d)
Positive mental well-being	40.1 (8.7)	47.1 (6.6)	6.5 (8.5)	–4.1 (27)	<.001	0.8
Self-efficacy^a^	4.8 (2.0)	5.9 (1.9)	1.1 (1.9)	–2.8 (26)	.01	0.5
Fatigue	5.9 (1.3)	5.5 (1.2)	0.4 (1.6)	1.2 (27)	.25	0.3
Loneliness	5.9 (2.0)	5.7 (1.9)	0.2 (1.8)	0.5 (27)	.60	0.1

^a^N=27 due to missing data.

**Table 5:** SWEMWBS^a^ scores across the program.

Measure	Week 1 score (N=19), mean (SD)	Week 4 score (N=16), mean (SD)	Week 7 score (N=16), mean (SD)	*F*_1.4,22.1_ value	*P* value
SWEMWBS	19.5 (4.5)	22.6 (4.9)	24.1 (4.6)	20.2	<.001

^a^SWEMWBS: Short-Form Warwick-Edinburgh Mental Wellbeing Scale.

### Goal Setting

Of the 47 participants, 26 (55.3%) used the goal-setting tool at least once, and a total of 97 goals were set across the 8-week course. The mean and median number of goals set by participants using the tool was 4 (range 1-9). All 97 goals were coded according to the 5 goal-oriented domains in TEDSS (see Table 6) [43]. Cohen κ was used to determine whether there was agreement between the 2 researchers’ judgement on whether participants’ goals were categorized as activity, internal, social interaction, health behavior, or disease-controlling strategies. There was good agreement between the 2 researchers (κ=.77, 95% CI 0.557-0.973, *P*<.001).

The goal-setting tool within the intervention was based on SMARTER goal setting. In personal communication, 1 participant emphasized the importance of setting *realistic* goals (eg, lowering expectations in line with what is possible now, not what was achievable pre–COVID-19). Further, the concept of *time* may be different depending on individual circumstances, symptoms, physical ability, lifestyle, etc, and goals are *measurable* in terms of improvement rather than being time oriented.

**Table 6:** Frequency of goal type in each domain of TEDSS^a^, with examples extracted from participant entries in the goal-setting tool embedded within the Hope Programme for Long COVID.

Domain	Goal type frequency (N=97), n (%)	Examples (“My goal is…”)
Activity strategies: finding ways to participate in everyday activities despite experiencing problems, such as fatigue, pain, or memory loss	47 (48.5)	“Plan my routine more each day…knowing what I am doing saves energy and reduces stress” [JH] “Going back to work for 2 hours a day…to feel useful again” [LG] “To get my house ready for Christmas…because I loved Christmas” [TC] “Tonight I am going to paint my toenails…because it makes me feel nice when I care for myself” [G] “I am going to begin singing lessons one day each week at a music studio…I want to surprise my daughter by singing at her wedding” [KW]
Health behavior strategies: maintaining a healthy lifestyle to enhance health and limit the risk of lifestyle-related illness	18 (18.6)	“To walk more than I have already done…to build up my strength and stamina” [AS] “Eat healthier snacks each day at work…what I eat can make a big difference to how I feel” [JH] “To continue eating healthy and losing weight…because I know my health and recovery will benefit” [VL] “To work on my breathing exercises more…it will help improve my general condition and health” [Ru]
Internal strategies: preventing and managing stress, negative emotions, and internal distress and creating inner calm	16 (16.5)	“To be positive and have a purpose in life…to conquer fear, it helps my panic attacks and nervous tension” [DS] “To increase mindfulness activities at home…switch my brain off and have a proper rest” [LT] “To have a relaxing day today…because everyone needs to unwind” [TC]
Social interaction strategies: managing social interactions and relationships to be able to participate without exposure to negative reactions	10 (10.3)	“To reach out to two people I haven’t spoken to for a while on Whatsapp…because I value their friendship” [AM] “To go our for lunch with my daughter on a Thursday lunchtime in a café…because I enjoy her company” [TC] “I want to make sure I keep in touch with my friends and family, by phone or in person…because these relationships are very important to me” [IA]
Disease-controlling strategies: preventing, controlling, and limiting symptoms, complications, or disease progression	6 (6.2)	“Starting a physical rehabilitation course…I so desperately want to get better” [SC] “To feel less dizzy by doing my exercises…it will make me feel better overall with long covid symptoms” [KB]

^a^TEDSS: taxonomy of everyday self-management strategies.

## Discussion

### Principal Findings

In this study, we aimed to codevelop and test a digital self-management intervention for people with LC and estimate the parameters for a full trial. Sixteen people with lived experience of LC (both patients and professionals) led the codevelopment phase through workshops and interviews to discuss unmet needs and areas in which they felt support was lacking. This allowed us to coproduce bespoke and relevant content aligned to the needs of many people with long-term effects of COVID-19.

Our target sample size of 40 was exceeded (N=47), suggesting the advertising campaign for enrollment and subsequent email invitation to participate in the study is a viable strategy. The postcourse questionnaire completion rate was suboptimal (60%), when compared to the >70% completion target in our previous Hope Programme for Long COVID study protocol [44] and 80% completion rates in other Hope Programme for Long COVID studies [20,22]. This will be addressed in future studies by enhanced incentives for completion (see later). Nevertheless, the postprogram outcome data indicated significant improvements in mental well-being and self-efficacy scores at the end of the program relative to the start. A change in the WEMWBS score of 3 or more points is a minimally important level of change, so an average group increase of 7 points from pre- to postprogram is extremely encouraging. However, this measure was taken immediately at the end of the intervention rather than at a later follow-up, so longer-term changes are yet to be determined. Furthermore, the finding that the most significant improvement in positive well-being is made within the first half of the course is essential for our planning and design of future interventions. For example, future courses may consider presenting modules in the first half of the intervention that participants rank as being the most useful or address the most common issues, such as fatigue. An alternative explanation could of course be that participants found the second half of the course to be less useful. Our next trial will incorporate follow-up interviews with participants and facilitators to gain a better understanding of their experience of the course and suggestions regarding the relevance of all modules.

The significant improvement in self-efficacy is promising, as the Hope Programme for Long COVID is a self-management course providing tools to support people to manage their own health and well-being. These early data indicate that participants feel more confident to manage their COVID-19–related symptoms after taking part in the course. However, we must exercise caution when interpreting these changes in participant scores, as the study was not powered to detect statistically significant changes. Full inferential analysis and statistical significance will be determined by a fully powered trial.

All goals set by participants mapped onto the 5 goal-oriented domains in TEDSS [43]. The most frequent type of goals related to activity strategies, such as using the 3 Ps (ie, pace, plan, prioritize), organizing time and routines, and engaging in meaningful activities. These relate to common self-management techniques and are embedded throughout the Hope Programme for Long COVID. It is encouraging that participants appear to be using these strategies in setting and planning their own short-term goals, giving further assurance of the usefulness of the intervention for self-managing LC symptoms. Interestingly, goals relating to disease-controlling strategies were the least common types of goals set by participants in this study. This is perhaps because of the varied nature of LC symptoms and a lack of clear medication management that would be inherent in other complex conditions. As medical and scientific research on treatments for LC continues, disease management strategies may become more applicable within everyday self-management of LC symptoms.

The Hope Programme for Long COVID was delivered digitally to allow participants flexibility in when and how they accessed the intervention. The wider literature reports this as the preferred format of participants seeking peer support [3,4]. Although we did not offer an in-person option, digital face-to-face meetings were well attended in the form of Hope Cafés. It is not possible to know whether an in-person delivery would have different outcomes, but we believe that digital delivery removes barriers introduced by physical ill-health (eg, fatigue) and time constraints (eg, employment, care duties) in the population with LC.

### Strengths and Limitations

A major strength of this study is the rapid development and deployment of the Hope Programme for Long COVID, due to the repurposing of an existing digital intervention for long-term conditions. The course was developed and delivered by a multidisciplinary team and social enterprise, exploiting existing networks to draw upon professional and clinical expertise and maximize the impact of the Hope Programme for Long COVID. A further strength of this study is the depth of understanding gained about how the intervention is used by people with LC. That is, the data showed that the most significant change in mental well-being is likely to occur in the first half of the intervention, indicating that the topics covered in the first 4 weeks of the intervention are the most useful for participants or possibly that 4 weeks of peer support are sufficient to enable a positive change in mental well-being. It may be that participants gain a validation of their condition and a feeling of universality after only a few weeks on the program. This could be especially valuable, given the experience some patients have had of being dismissed by health professionals and stigmatized or invalidated socially [5-7]. Goals were most frequently related to activities and health behaviors, indicating that the motivations, priorities, and needs of participants center around staying physically and mentally healthy through planning meaningful activities. It must be noted that this was a self-selecting sample, where participants who saw the ads were motivated to join the intervention and the research. Improvements in mental well-being and self-efficacy may be inflated by scores from people who were simply more open to, or actively searching for, self-management support.

This study yielded encouraging data regarding improved mental well-being and self-efficacy scores after the course. This pre-post study was not powered, a priori, to detect a statistically significant difference in key outcome measures, nor to account for potential covariates, such as age or education, which would ideally be included in a multivariate model to test the effect of the intervention. Nevertheless, we presented preliminary inferential statistics to signal efficacy. For a study of this nature to be sufficiently powered, based on the effect sizes noted within this study, and retaining an α error rate of .05, a (1 – β) error of .95, and a 2-tailed hypothesis, we would need to recruit 32 participants based on the WEMWBS [35], 54 participants based on the Self-Efficacy in Managing Chronic Disease scale [36], 147 participants based on the FSS [38], and up to 1302 participants based on the UCLA Loneliness Scale [40]. (Note that these sample sizes are presented assuming each variable to be to primary outcome.) In a future study, the inclusion of a control group would allow comparison of postcourse scores between those who took part in the Hope Programme for Long COVID and those who had not received the intervention. A fully powered study using the WEMWBS as the primary outcome measure would require 87 participants per group (ie, intervention and control groups) to minimally detect moderate effect sizes (ie, Cohen f≥0.25, Cohen d≥0.5) or 42 participants per group to minimally detect large effect sizes (ie, Cohen f≥0.4, Cohen d≥0.8), both with a power of 0.95, and an a priori α of .05 [45]. The results of the next study—a waitlist control trial—will allow us to specify with greater confidence that improvements in well-being and self-efficacy are related to the intervention, rather than natural, spontaneous recovery over 8 weeks. Collecting longer-term follow-up data in the next study will show whether any improvements continue beyond the end of the intervention and whether participants are making use of the techniques and self-management strategies months later.

Participant interviews will be incorporated into the research design of the next trial to capture important data regarding acceptability, nonusage, or reasons for dropout. These rich, qualitative data would help us understand the key factors behind participant “improvements” (eg, whether it was peer support, particular activities, or goal setting that people found most useful). Such data may also indicate why greater changes in well-being are observed in the first half of the course. Better incentives (ie, a guaranteed £10 (US $10.57) voucher for completing pre and postprogram surveys) will also be included to encourage participants to complete follow-up questionnaires regardless of whether they used or enjoyed the program.

### Conclusion

We codesigned a bespoke, digital self-management intervention with and for people with LC. Our recruitment rate was extremely encouraging, and the sample characteristics largely mirrored those of the wider UK population with LC. We enlisted an enhanced incentive strategy to improve completion rates of follow-up questionnaires in future research. We have already started our next trial (ISRCTN: 11868601) using a nonrandomized waitlist control design to further examine intervention efficacy.

## Abbreviations

CWR: Coventry, Warwickshire, and Rugby
FSS: Fatigue Severity Scale
H4C: Hope For The Community
LC: long COVID
NHS: National Health Service
SMART: specific, measurable, achievable, relevant, and timely
SMARTER: specific, measurable, achievable, relevant, time-based, enjoyable, rewarded
SWEMWBS: Short-Form Warwick-Edinburgh Mental Wellbeing Scale
TEDSS: taxonomy of everyday self-management strategies
UCLA: University of California, Los Angeles
WEMWBS: Warwick-Edinburgh Mental Wellbeing Scale
